# Decontamination Efficacy of Three Commercial-Off-The-Shelf (COTS) Sporicidal Disinfectants on Medium-Sized Panels Contaminated with Surrogate Spores of *Bacillus anthracis*

**DOI:** 10.1371/journal.pone.0099827

**Published:** 2014-06-18

**Authors:** Jason M. Edmonds, Jonathan P. Sabol, Vipin K. Rastogi

**Affiliations:** 1 U.S. Army - Edgewood Chemical Biological Center, Research, Development and Engineering Command, Aberdeen Proving Ground, Maryland, United States of America; 2 EXCET, Inc., Springfield, Virginia, United States of America; University of Minnesota, United States of America

## Abstract

In the event of a wide area release and contamination of a biological agent in an outdoor environment and to building exteriors, decontamination is likely to consume the Nation’s remediation capacity, requiring years to cleanup, and leading to incalculable economic losses. This is in part due to scant body of efficacy data on surface areas larger than those studied in a typical laboratory (5×10-cm), resulting in low confidence for operational considerations in sampling and quantitative measurements of prospective technologies recruited in effective cleanup and restoration response. In addition to well-documented fumigation-based cleanup efforts, agencies responsible for mitigation of contaminated sites are exploring alternative methods for decontamination including combinations of disposal of contaminated items, source reduction by vacuuming, mechanical scrubbing, and low-technology alternatives such as pH-adjusted bleach pressure wash. If proven effective, a pressure wash-based removal of *Bacillus anthracis* spores from building surfaces with readily available equipment will significantly increase the readiness of Federal agencies to meet the daunting challenge of restoration and cleanup effort following a wide-area biological release. In this inter-agency study, the efficacy of commercial-of-the-shelf sporicidal disinfectants applied using backpack sprayers was evaluated in decontamination of spores on the surfaces of medium-sized (∼1.2 m^2^) panels of steel, pressure-treated (PT) lumber, and brick veneer. Of the three disinfectants, pH-amended bleach, Peridox, and CASCAD evaluated; CASCAD was found to be the most effective in decontamination of spores from all three panel surface types.

## Introduction

In 2001, a number of letters containing *Bacillus anthracis* spores, the causative agent for the deadly anthrax disease, were processed and delivered to their respective recipients by the United States Postal Service resulting in contamination of several building interiors, including U.S. Postal & Distribution Centers in Brentwood, DC, Trenton, NJ, Hart Senate Office Building, Washington DC, and American Media Inc., Boca Raton, FL [1]. Despite heavy contamination levels of several building interiors, remediation of building interiors was achieved successfully by fumigation with chlorine dioxide (CD) or vaporous hydrogen peroxide (VHP) [2], [3], [4]. A number of solution based sporicidal disinfectants have been approved by U.S. EPA’s Office of Pesticides Programs, but were not used to great extent because their efficacy has been proven in laboratory-scale studies only.

Three standardized test methods (ASTM 2197-02, ASTM 2414-05, AOAC *Official Method* 2008–05) are available for determining the efficacy of sporicidal disinfectants and gaseous fumigants under pristine laboratory conditions [5], [6], [7]. These standardized test methods often use small (<1-cm) size test carriers of smooth, non-porous, and hard surfaces, such as steel and glass. Consequently, such methods are not suited for conducting efficacy studies of biologically contaminated wide area urban environments which include building structures composed of a vast array of both porous and non-porous materials. Furthermore, the current test methodologies rely on complete submersion of the inoculated coupons in test chemical, or use of adequate volume of test chemical to completely cover the contaminated surface during the test of liquid disinfectants. In the field, neither the test conditions are idealized, nor is the immersion of contaminated vertical (5, 6) or complex surfaces a possibility. Although a number of gaseous decontamination technologies have been investigated within large volumes of air within buildings [7], [8], [9], [10], [11], the application of gaseous technologies to outside surfaces of buildings over a large urban area would likely be challenging. Therefore, the assessment of liquid based decontamination technologies over large areas is relevant to large scale biological remediation.

Since adequate test methodologies [7] are lacking for field assessment, efficacy data on commercial of-the-shelf (COTS) sporicidal agents in decontamination of complex scale surfaces of mitigation and remediation potential is limited. Under the auspices of Interagency Biological Restoration Demonstration (IBRD), a federally funded program with the goal to reduce the time and resources required to recover and restore wide urban areas post-environmental incident, this study was initiated to generate quantitative efficacy data which could be extrapolated to large wide area decontamination attempts. Specific aspects of appropriate methodology included use of mid-size panel assembly (1.2 m^2^), spore inoculation and their sampling, decon application, sample concentration and spore enumeration.

Depending on the surface composition and the decontamination technology tested, the ability to recover viable spores from the panels after decontamination trials varied. Even though, a correlation between lab-scale assessment and field remediation is lacking with respect to post-decontamination spore recovery, related approaches and data is highly desirable. Restoration of buildings for occupancy is a very complex issue and requires a high degree of public trust in federal agencies authorized in declaring areas decontaminated with near-zero risk of infection. Buildings are composed of highly complex and porous surfaces which potentially pose a long-term and/or recurring threat to occupants and passers-by. The ability to efficiently and effectively decontaminate porous materials to safe levels is of great concern to officials charged with deeming a structure safe for re-occupancy after a contamination has occurred. Method flow charts and efficacy data from experiments using Peridox, pH-amended Ultra Clorox Germicidal bleach, and CASCAD, in decontamination of mid-size panels are presented in this paper.

## Methods and Materials

### Test Inoculum

Ten gram of *Bacillus atrophaeus* subspecies *globigii* Dugway 1088 batch 040 spores (BG spores) were washed with cold water (4+2 C), pelleted using centrifugation at 5000×g for 30 minutes, and resuspended six times in 50 mL cold sterile distilled water. After the final wash, spores were suspended in 50 ml of de-ionized sterile water and the master stock with a spore titer of 1.5×10^1^°Colony Forming Units (CFU)/mL was enumerated, by serial dilution plating. The stock was stored at 4°C until used within 30 days. Spore stocks were periodically checked by performing the Schaeffer-Fulton spore staining procedure to confirm the spore integrity and spore:vegetative cells ratio. The spore:vegetative cells percent ratio was 80∶20. The stock was heat-shocked at 65°C for 30 minutes before use to render all vegetative cells non-viable. Working stocks with a titer of heat-resistant 1.5×10^9^ CFU/mL were prepared by appropriately diluting the master stock with sterile water and confirmed by serial dilution and enumeration on tryptic soy agar (TSA) plates.

### Panel Construction

Panels of stainless steel, PT (pressure-treated) lumber, and brick veneer were assembled in 1.2 m×1.2 m size. All panels were constructed with 1.1 cm thick 1.2 m×1.2 m oriented strand board (OSB) as a backing material (Home Depot, Cat. No. 386-081). Stainless steel (Durrett Sheppart Steel, Baltimore, Maryland) panels were composed of eight individual sheets of 30.5 cm×30.5 cm in size. The sheets of T-304 No. 2B finish 20 gauge stainless steel sheets were glued to the OSB backing board with construction adhesive to form a single four 1.2 m by 1.2 m panel. PT lumber (Home Depot, Cat. No. 155-400) panels were constructed by assembling 8 boards measuring 1.2 m in length, 14 cm in width, and 1.1 cm in thickness as well as one board measuring 1.2 m in length, 10.2 cm in width, and 1.1 cm in thickness to achieve a PT lumber panel of desired size. The PT lumber was secured to the OSB with a single 3.2 cm exterior screw (Home Depot Cat. No. 131-537) at each end of the board. Brick panels were constructed per manufacturer instruction by securing a metal grid (Brickit.com, Bohemia, NY, Cat. No. MGMOD48x8) to OSB panels with construction adhesive and 1.3 cm exterior screws. 1.3 cm think brick veneer (Brickit.com, Bohemia, NY, Cat. No. TSMODKINGW) was then secured to the metal grid using construction adhesive.

### Panel Inoculation

**Figure 1:**
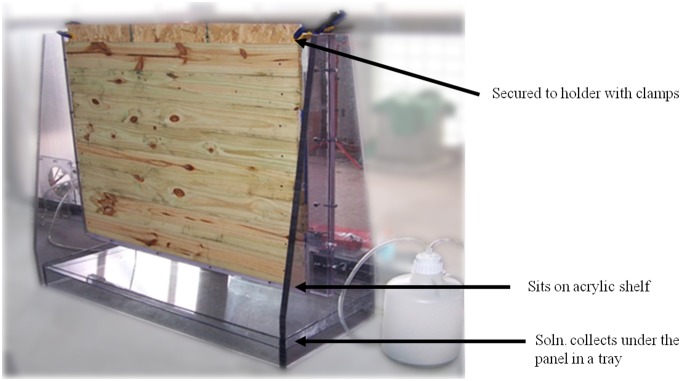
Sampling flow chart.

Panels constructed for decontamination testing were inoculated with 1,280 individual 10-µL drops of previously described working stock of BG spores evenly distributed throughout the panel with the use of an electric micro-pipettor to achieve a final calculated total spore load of ∼2.1×10^9^ spores per panel based on previous suspension enumeration. Inoculation was done in a preparation area outside of the 81 m^3^ ambient decontamination chamber and after inoculation, moved into the chamber and placed onto panel holders as described below. Because temperature and RH within the chamber are subject to the surrounding conditions, the panels were set aside to allow the spore suspension to dry for 24 h. Only the required number of panels to be tested the following day were inoculated with spores at any given time, and used within 48 h of inoculation. Panels were designated as control panels or sample panels and underwent treatment as detailed in Figure 1. Due to the large size of the panels, we were unable to sterilize each panel prior to testing. However, controls were taken periodically during testing to confirm that panels were not being contaminated with spores from natural flora nor testing procedures (data not shown).

### Application of Decontamination Technologies

**Figure 2:**
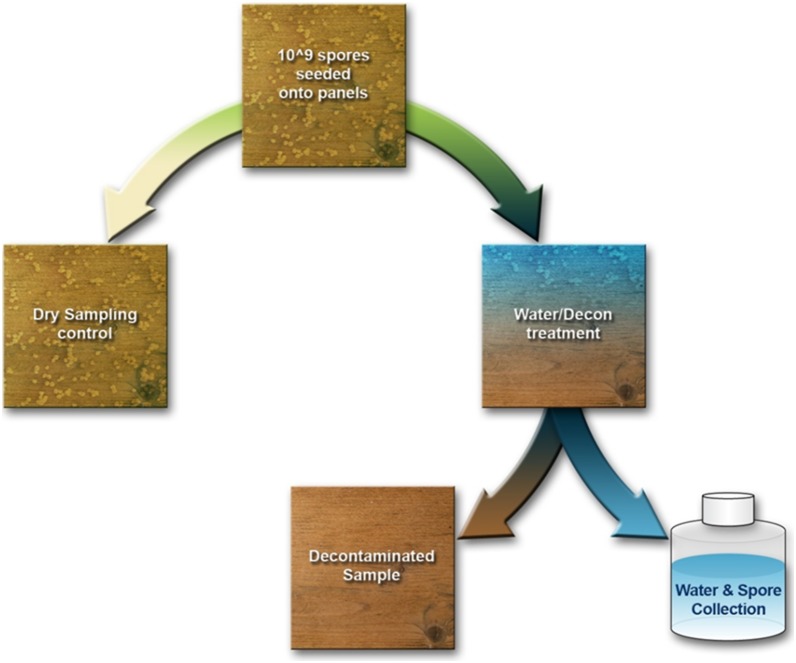
Panel holder for decontamination application.

Inoculated panels were attached vertically to a specially constructed panel holder (designed by Dr. Rastogi and fabricated by the Advanced Design & Manufacturing Team at the Aberdeen Proving Grounds, Edgewood Area, APGEA) with a wood clamp in both the upper right and upper left corners. The run-off from the subsequent application of the decontamination solution was collected at the bottom (Figure 2). Contaminated panels were treated with one of three disinfectants, Peridox, a peroxide technology, (Clean Earth Technologies, Earth City MO, Cat. No. Per-1), 1∶10 pH amended Ultra Clorox Germicidal bleach (pH 7+0.1, as described in Tomasino *et al*., 12), and CASCAD, a combination of peroxide and hypochlorite (Allen Vanguard Technologies, Ottawa, ON, Cat. No. GP2100-730, GCE2000-950, and GPX-4000). Peridox and CASCAD were diluted per manufacturer’s directions. In addition, the control panels were sprayed with water to assess recovery of the inoculated spores. Each disinfectant was filled into a low pressure 15.1 L backpack sprayer (Agri Supply Co., Garner, NC, Cat. No. 59540) and the respective decontaminant (or water in the case of control panels) was sprayed on to the appropriate panels from a distance of ∼46 cm. Panels were visually monitored to ensure that they remain wet for a contact time of 30 min with the decontamination solutions. Panels were allowed to become visually dry by standing in the vertical position for 2 h prior to sampling.

After an initial decon application, the panels were set aside for two weeks, during which time no panels were sampled. Panels were then subsequently treated with a reapplication of the respective decontaminant. The only exception was with CASCAD where half of the panels received a reapplication of CASCAD solution while the other half were rewetted with distilled water. Post-reapplication sampling of the panels was performed identically as described for the initial application.

### Sampling Stainless Steel Panels

Stainless steel panels were sampled in 30.5 cm by 30.5 cm sections with each section sampled using 1/8 polyurethane wipe (VWR, Inc., Bridgeport, NJ, SterileWipe* LP Wiper, ITW Texwipe*, Cat. No. TWTX3211). Sixteen pre-wetted wipes were used per panel. Each wipe section was folded into fourths providing four wiping surfaces, with each surface used on the same 30.5-cm^2 ^section of stainless steel panel. Each panel was wiped ten times along the width, refolded to expose a clean wipe surface, and then wiped 20 times along the length. This procedure was repeated an additional time for a total of four individual wiping events per panel. After wiping, the wipes were placed into individual 50-ml conical tubes each containing 20-ml of recovery solution, PBST (phosphate saline buffer, pH 7.4 containing 0.04% Tween-80). The 16 wipes were processed as individual samples and subsequently the data was pooled. The tubes containing polyurethane wipes, were vortexed for ten minutes using a large capacity mixer (Glas-Col; catalog no. 099A-LC1012; Terra Haute, IN). After vortexing, the tubes were sonicated in a sonic bath (Branson 5510; Branson Ultrasonics Corporation, Danbury, CT) for an additional ten minutes. After spore extraction, the samples were plated in triplicate, using a spiral-plater (Spiral BioTech Autoplate 4000; Advanced Instruments, Norwood, MA). Plates were incubated over-night at 37°C and colonies were counted the next day using a Q-Count instrument (Advanced Instruments, Norwood, MA). The recovered CFUs were recorded and Coefficient of Variations (C.V.s) calculated. The total CFUs estimated per panel were compared to the spore number inoculated and percent recovery was calculated as follows: number of spores recovered/spore number inoculated ×100.

### Sampling Brick and Lumber Panels

The two porous surface materials, PT lumber and brick, were sampled with a vacuum sock technology (Midwest Filtration co, Cincinnati, OH, vacuum filter sock collection kit, and Omega HEPA Vacuum, Cat No. FAB-20-01-001A and 950-A1-00-120). Each panel was sampled with a single vacuum sock. The nozzle of the collection tube was placed approximately 1.3 cm above the surface. The nozzle was slowly moved back and forth across the surface using left-to-right horizontal strokes to collect spores. This procedure was repeated two more times touching the nozzle to the surface of panel and using top-to-bottom vertical strokes and left-to-right horizontal strokes. The nozzle was removed from the vacuum hose and vacuum sock removed from the filtration nozzle. The vacuum sock filter was first cut and then placed into 50 mL conical tubes containing 35 mL PBST and pushed down to submerge the filter in the fluid. The spores were extracted from the sock filter after vortexing and sonication as described above.

### Sampling Liquid Run-off

Run-off samples were collected from the collection tray under the panel stand. Both test and control panels were maintained wetted for 30 min by repeated sprays. The runoff sample volumes were collected, measured, recorded, and aliquots were serially diluted and plated as described above. Because of the large runoff volumes collected, the samples from test panels were expected to be dilute. Therefore, in order to keep the detection limit low, a 25 mL aliquot of the run-off sample from the collection tray was filtered through a 0.2 µm syringe filter. The filter was then rinsed twice by passing 25 mL of sterile distilled water through the filter. Filters were then placed into conical tubes with extraction buffer and processed as described above. In a separate study, the spore release efficiency from the filter was determined to be 60–80% (data not shown).

### Sampling Analysis, Data Handling, and Statistical Treatment

Samples from each of the 16 wipes used to sample stainless steel were enumerated and the data was pooled for each panel. The vacuum filters used to sample lumber and brick, were serially-diluted and plated in triplicates, and the mean of each triplicate recorded. Mean colony-forming units (CFU) counts for each data set were calculated by averaging respective run-off and surface sampled spores. The total CFU numbers were transformed into log10 values. Since three experimental runs, each with three panels were performed, the log(CFU) values were averaged (mean of the logs) and SD values calculated. The log reduction (LR) values were computed by subtracting log(CFU) values from treated panels from that from control panels. For percent recovery (%RE) values were calculated by dividing the mean recoverable CFUs from the sampling material from control panels by total number of spores inoculated onto the panels. Control panels were treated just like test panels, with the exception that water was used in place of disinfectant, and the spore recovery was performed in triplicates.

## Results

### Sampling Recovery Efficiency

Polyester wipes were used to sample spores off steel panels and vacuum socks were used to sample spores from the other two porous panel types. Recovery efficiencies of sampling technologies were estimated by the number of viable spores recovered from control panels which had not been sprayed with any solution. Greater than 9.2 logs of spores were recovered from the stainless steel panels, which represented approximately 76% of the spores inoculated onto the panels (Table 1). Recovery efficiencies from brick and lumber were significantly lower, approximately 7.3 and 7.5 logs, respectively, which accounted for <1% of the spores inoculated onto these panels.

**Table 1:** Relative spore recovery from untreated panels.

Panel Type	% Recovery	Log Spores Recovered (SD)
Steel	76	9.2+/−(0.3)
Brick	1	7.3+/−(1.5)
Lumber	1	7.5+/−(1.8)

n = 3.

### Control Spores Collected in Runoff

Inoculated panels were sprayed with water to estimate mechanical spore removal from each panel type. Approximately 8.7 logs were recovered in the water runoff from stainless steel panels, which represented 24% of the spores inoculated onto the panels. (Table 2). From the other two panel types, brick and lumber, approximately 8.2 logs or 8%, and 8.6 logs or 16% of the spores inoculated onto the panels were respectively recovered in the water runoff (Table 2).

**Table 2:** Spores recovered in the runoff.

Panel Type	% Recovery	Log Spores Recovered (SD)
Steel	24	8.7+/−(0.4)
Brick	8	8.2+/−(1.1)
Lumber	16	8.6+/−(0.3)

n = 3.

### Efficacy of Decontamination Technologies

**Figure 3:**
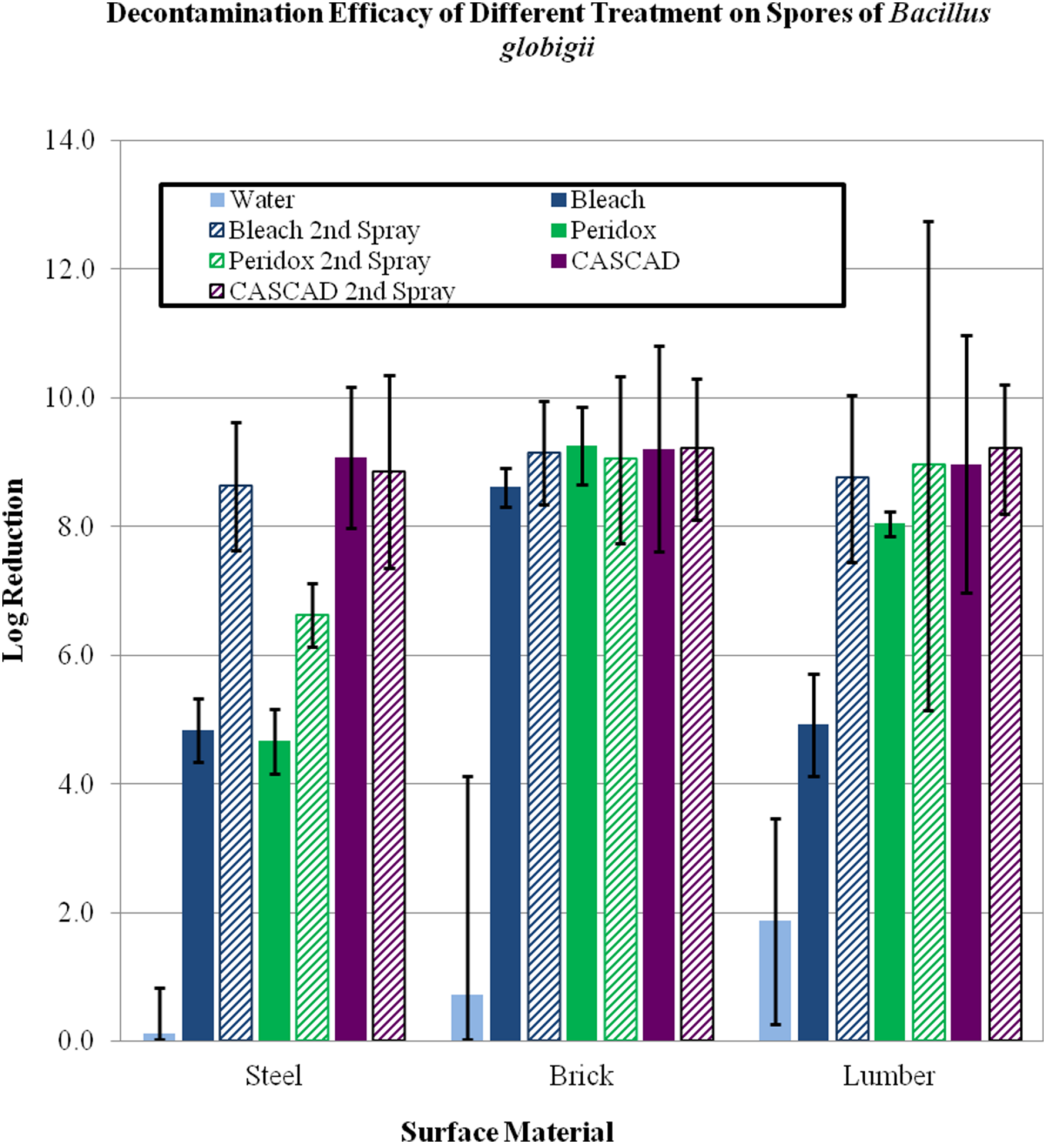
Sporicidal efficacy of three COTS disinfectants.

All panel types were treated with two applications of decontamination solution. The first application of decontamination solution on stainless steel panels resulted in 4.8, 4.7, and 9.1 log reduction (LR) in number of viable spores when treated with Ultra Clorox Germicidal bleach, Peridox, and CASCAD respectively (Table 3, Table 4, Figure 3). After the second application, the LR values increased to 8.6 (with Bleach), and 6.6 (with Peridox), and remained constant at 8.9 with CASCAD with no statistical difference between the three technologies (Table 4, Table 5, Fig. 3). With the exception of Peridox, two decontaminant applications result in comparable LR values on stainless steel panels (Table 4).

**Table 3:** Log10 reduction after initial decon application of decontamination solution (SD*).

Panel Type	Disinfectant Type Applied			
	Water	Bleach	Peridox	CASCAD
Steel	0.1+/−(0.7)	4.8+/−(0.5)	4.7+/−(0.5)	9.1+/−(1.1)
Brick	0.7+/−(3.4)	8.6+/−(0.3)	9.3+/−(0.6)	9.2+/−(1.6)
Lumber	1.9+/−(1.6)	4.9+/−(0.8)	8.0+/−(0.2)	9.0+/−(2.0)

* = Standard deviation, **n = 9**.

**Table 4:** Pair-wise Efficacy Analysis of Decontamination Technologies.

First application of Decontamination Technology			
	Stainless Steel	Brick	Lumber
Bleach			
Peridox	NSD	NSD	p<0.005
CASCAD	p<0.05	NSD	NSD
Peridox			
CASCAD	p<0.1	NSD	NSD
**Second application of Decontamination Technology**			
	**Stainless Steel**	**Brick**	**Lumber**
Bleach			
Peridox	p<0.1	NSD	NSD
CASCAD	NSD	NSD	NSD
Peridox			
CASCAD	p<0.2	NSD	NSD
**Reapplication of Decontamination Technology**			
	**Stainless Steel**	**Brick**	**Lumber**
Bleach	p<0.05	NSD	p<0.05
Peridox	NSD	NSD	NSD
CASCAD	NSD	NSD	NSD

All decontamination technologies, when compared to water, resulted in p<0.0001; *NSD = No statistical difference.*

**Table 5:** Cumulative log10 reduction after reapplication of decontamination solution (SD*).

Panel Type	Disinfectant Type Applied		
	Bleach	Peridox	CASCAD
Steel	8.6+/−(1.0)	6.6+/−(0.5)	8.9+/−(1.5)
Brick	9.1+/−(0.8)	9.0+/−(1.3)	9.2+/−(1.1)
Lumber	8.7+/−(1.3)	8.9+/−(3.8)	9.2+/−(1.0)

* = Standard deviation, **n = 9**.

The sporicidal efficacy of all three decontaminants on two porous surfaces, PT lumber and brick, was comparable (LR values of 8.7, 8.9, and 9.2) after second application (Table 4), even though the LR value was significantly lower for bleach on PT lumber after the first application. (Table 3, Table 4, Table 5, and Figure 3).

## Discussion

Biological sampling and recovery from environmental surfaces is a complex issue, and is typically in the range of 5–60%, especially when inoculated as liquid suspension (7, 12). The spores inoculated on surfaces get partitioned three ways. One, a fraction of spores is irretrievable due to spore lodging into the pores and spore adhesion to the surface matrix. Second, a fraction of surface remains on the sampling tool surfaces, i.e. wipes or vacuum socks. Finally, the third fraction, which is retrieved from the surface by the sampling tools and those released from such sampling matrices. Sporicidal efficacy is determined from the fraction of spores that are accountable in the third fraction, resulting in not accounting for those in the other two fractions. These sampling limitations suggest that additional studies are needed to improve spore recovery by sampling tools.

A methodological approach with the goal of providing operational testing in the context of consequence management following a wide-area release and to assess the sporicidal efficacy of three COTS disinfectants, Ultra Clorox Germicidal bleach, Peridox, and CASCAD, are summarized in this paper. Even though the manufacturer’s recommended contact times are ≥30 min, it is unreasonable and unrealistic to expect that the vertical surfaces be kept wetted for this long of a period of time in a large area environment. Approximately, 7.6 L of decontaminant was sprayed to ensure a contact time of 30 min for bleach and Peridox. Respraying was performed, every 2–5 min depending on the temperature and RH on a given test day, even though it may be unrealistic in ‘real-life’ scenario. With CASCAD, approximately 5.7 L per panel was used, and required only a single reapplication. This was due to the foaming/sticking properties of this decontaminant. Typical temperature and RH at the time of testing in June through August were >80% RH and >27°C. Both these physical parameters affected the total volume and the number of times, a given decontamination technology was applied.

With one 30 min application, CASCAD outperformed the other two decontaminants on stainless steel panels and significantly outperformed bleach on lumber panels. This result was not surprising as decontamination attempts using bleach on pinewood has previously been reported as ineffective [12]. The chemical composition of PT lumber can neutralize the active OCl^−^ species in bleach. On brick, however, all three decontamination technologies performed similarly, which varies with the previously reported performance of Peridox on brick [13]. The Peridox performance discrepancy could in part be due to the type of brick (and the components) used in the two studies. On steel panel, quick run-off of Peridox solution from the smooth vertical surface could have resulted in poor contact times leading to poor performance.

Although both brick and PT lumber are porous materials, and the stainless steel panels maintained their integrity after application of decontamination technology and did not corrode, the effectiveness of bleach to decontaminate these materials greatly differed while that was not true for Peridox and CASCAD suggesting that porosity alone is not responsible for decontamination efficacy (Table 4). It is likely that CASCAD outperformed the other two decontamination technologies due to the foaming, greater adhesion to the surfaces, and/or a higher chlorine content (10 fold higher concentration when following manufacturer’s recommendation) compared to the Ultra Clorox Germicidal bleach solution.

While the efficiency and efficacy of the different decontamination technologies varied based on the technology used and the surface treated, sample to sample variation was a common underlying observed phenomenon. For each of the initial technology and surface combination, 10 out of 12 combinations produced CVs>50%, and with the re-sprays, only one combination resulted in a CV<49%. The high CV value is an indication of the potential difficulty in assessing and achieving consistent and efficient large-scale decontaminations. The variation in recovery of viable spores could result from a combination of factors, such as of the size of the panel (1400x larger than typical coupon size of 10-sq-cm), method of inoculation (suspension inoculated as small droplets), and errors associated in consistent application of decontamination technology, and most importantly variations in sampling of large panels.

One notable source of error in this study was the use of the vacuum socks technology for spore recovery from porous materials. A 99% reduction in the number of spores recovered from the porous materials without use of decontaminants documents a significant reduction in spore recovery with the use of current technology. The inconsistent results obtained from this sampling technology suggests limitations for environmental sampling applications. A study by Brown, *et al*. (2007) evaluated the vacuum filter sock and has identified several characteristics, including pore diameters of over a micron, contributing to the inefficiency of this particular sampling device [14].

In addition to a number of factors affecting sampling efficiencies, inherent characteristics of the surface materials, including porosities, chemistry, and the effects of spore surface composition on adhesion forces to a given surface type are not well understood [15]. A large gap exists with respect to our understanding in how varying porosities of surface material might protect spores from decontaminants. Additionally, if the biological agent is applied to porous materials as a wet aerosol, or applied to a wet surface, or comes into contact with rain prior to decontamination applications, the number of spore in the water run-off from the matrix is unknown and would likely over-estimate LR values [16]. In control experiment in which water was sprayed onto the panels, only 9% of the spores deposited onto the brick and 17% of the spores deposited onto the lumber panels were accounted for in runoff or by vacuum sampling as contrasted to near 100% mass balance accountability from the stainless steel panels. Current capability to estimate the penetration of agent into porous surfaces such as brick and lumber is lacking. The authors acknowledge that the recovery data presented here is a reflection of a number of factors which influence the ability to sample, recover, and culture spores. While the number of spores recovered using the current sampling technology for porous materials and the number of spores collected in the run-off have been quantified, those embedded within the matrix of the brick and lumber after sampling trials is unknown but does contribute to the number of available recoverable spores reported in this study.

Additionally, even though the panels were sampled while visually dry, the impact of retained moisture within the porous matrix of brick and lumber on spore recovery of is unknown. An improvement in vacuum-based or other porous material sampling devices as well as a fundamental understanding of effects of adhesive forces on physical interaction of bio-agent with surface materials is critical to improving the recovery efficiency and decontamination efficacy assessment of wide-area response and recovery efforts.
